# Spatial Distribution and Migration Characteristics of Heavy Metals in Grassland Open-Pit Coal Mine Dump Soil Interface

**DOI:** 10.3390/ijerph19084441

**Published:** 2022-04-07

**Authors:** Zhen Cai, Shaogang Lei, Yibo Zhao, Chuangang Gong, Weizhong Wang, Changchun Du

**Affiliations:** 1 School of Public Policy & Manage, China University of Mining & Technology, Xuzhou 221116, China; zhen.cai@cumt.edu.cn; 2 Engineering Research Center of Ministry of Education for Mine Ecological Restoration, China University of Mining & Technology, Xuzhou 221116, China; tb19160018b2@cumt.edu.cn; 3 School of Environment and Spatial Informatics, China University of Mining & Technology, Xuzhou 221116, China; 4 School of Spatial Informatics and Geomatics Engineering, Anhui University of Science and Technology, Huainan 232001, China; gongchuangang@126.com; 5 Inner Mongolia Zhungeer Banner Mining Area Career Development Center, Ordos 010399, China; wvz123888@163.com (W.W.); zqbcb@163.com (C.D.)

**Keywords:** open-pit coal mine dump, heavy metal, soil interface, spatial distribution, hyperspectral measurement

## Abstract

The open-pit coal mine dump in the study area contains many low-concentration heavy metal pollutants, which may cause pollution to the soil interface. Firstly, statistical analysis and geostatistical spatial interpolation methods described heavy metal pollution’s spatial distribution. The mine dump heavy metal pollution distribution is strongly random due to disorderly piles, but it is closely related to slope soil erosion. Furthermore, the soil deposition area is where pollutants accumulate. For example, all heavy metal elements converge at the bottom of the dump. Usually, the pollution in the lower part is higher than that in the upper part; the pollution in the lower step is higher than the upper step; the pollution in the soil deposition locations such as flat plate and slope bottom is higher than the soil erosion locations such as slope tip and middle slope. Finally, the hyperspectral remote sensing method described heavy metals pollution’s migration characteristics, that the pollutants could affect the soil interface by at least 1 km. This study provides a basis for preventing and controlling critical parts of mine dump heavy metal pollution and pollution path control.

## 1. Introduction

Open-pit mining significantly impacts land resources and the ecological environment [1]. The largest artificial structure in the open-pit coal mining area is the mine dump formed by stripping off the overlying topsoil and strata of the coal seam and then stratifying and stacking due to open-pit mining, which will cause land occupation, soil erosion, topographic and geomorphological damage, and biogeochemical process change [2,3,4]. Heavy metal pollution has always been a global issue. On the one hand, the enrichment of heavy metals makes them difficult to degrade in the environment [5]. On the other hand, reaching a specific concentration of heavy metals will negatively affect living things [6]. For heavy metal pollution caused by mining wastes such as gangue dumps and tailing ponds, and the primary environmental problems it causes, there have been systematic studies and perfect solutions from different perspectives [7,8]. In contrast, there is a lack of research on heavy metal pollution in open-pit coal mine dumps. Under the long-term catalysis of permanent storage and particular climates, such as the rainy season, heavy metals in mine dumps spread to the surrounding atmosphere, water, and soil systems through weathering, erosion, and seepage, posing a threat to the ecosystem and public health. Therefore, it is necessary to pay attention to this issue [9,10,11].

In most previous studies on heavy metal pollution in mining, solid mine dumps use gangue dumps and tailing ponds as the research objects [12,13,14]. Due to the homogeneity of materials, previous studies prefer to use spatial interpolation methods based on laboratory chemical analysis and geographic information systems (GIS) to identify the spatial distribution of pollution in surrounding soil, water, and sediment, thereby determining the migration trend of heavy metals and potential risk areas [15,16,17,18]. It is of great significance and necessity to master the pollution distribution law of the mine dump soil interface with significant material heterogeneity, rather than just staying in the surrounding areas. Furthermore, the traditional method of extracting the distribution of heavy metals in the soil has some shortcomings, such as a high cost, a time-consuming process, and an inability to monitor large areas quickly. Given this, hyperspectral remote sensing emerged [19,20]. This study attempts to use large-scale regional aerial remote sensing images to characterize the distribution of heavy metals in the soil interface surrounding mine dumps, aiming to explore whether this method is equally effective for low-concentration mine dump heavy metal pollution. Correspondingly, the evaluation methods applied to mining heavy metal pollution are accurate and diverse, including the Nemerow comprehensive index method, the enrichment factor method, geo-accumulation index method, pollution load index method, potential ecological harm index method, and human health risk method [16,21,22]. However, there are few direct evaluations on identifying stratum waste, the primary source of heavy metal pollution in mine dumps, and the potential source of heavy metal pollution in mining areas.

**Figure 1:**
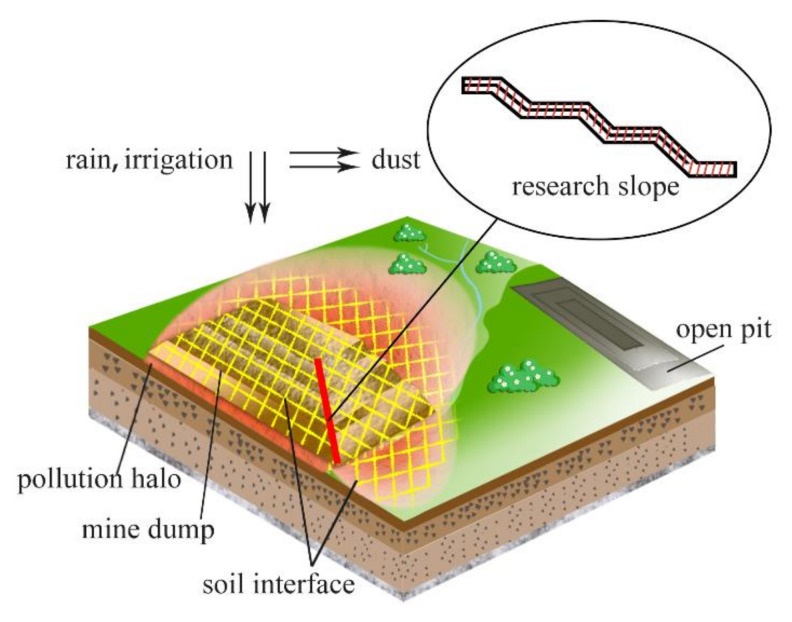
Schematic diagram of migration of heavy metals in grassland open-pit coal mine dump.

As one of China’s 14 large coal-electricity bases, the Shengli coal-electricity base is located in the Xilinguole grassland, a substantial agricultural and livestock product base in China, and a critical northern sand prevention belt for protecting ecological security [23,24]. Due to the ecologically fragile local environment and the lack of natural soil and mine dump topsoil, the continuous leaching of wind erosion and precipitation irrigation will cause heavy metals to migrate and diffuse with the soil and water, then accumulate in the surrounding environment and ecosystem [25,26]. The soil interface is the primary material exchange channel between the mine dump and the external environment and the indispensable path for internal heavy metals to migrate outside [27]. Therefore, the distribution pattern of heavy metals at the soil interface is significant to mine dump vegetation selection and slope optimization (Figure 1). Relevant studies on the spatial distribution and sources of heavy metals in the topsoil of this mining area have established that the average concentration of heavy metals Cu, Cd, Pb, Se, As, Cr, and Sn in the soil of the mining area generally exceed the background value, and the mine dump is one of the primary sources of heavy metal pollution [28]. Through the investigation of the overlying strata of the coal seam, namely mine dump materiel, in the mining area, it can be found that there are different levels of heavy metals exceeding the standard, which deserves further attention [29].

This research utilizes geographic information systems (GIS), hyperspectral remote sensing, numerical simulation, and statistical methods. The main objectives are as follows: (1) determine and evaluate the pollution degree and risk of heavy metals in mine dump soil interface; (2) determine the specific polluted location by analyzing the spatial distribution characteristics of heavy metal pollutants in dump soil interface; (3) determine the pollution range through the analysis of the spatial distribution of heavy metal pollutants in the soil interface around the mine dump. Based on the pollution distribution characteristics obtained above, some prevention and control suggestions are proposed. This study provides theoretical and technical support for preventing and controlling the open-pit mine dump heavy metal pollution and land reclamation.

## 2. Materials and Methods

### 2.1. Study Area

**Figure 2:**
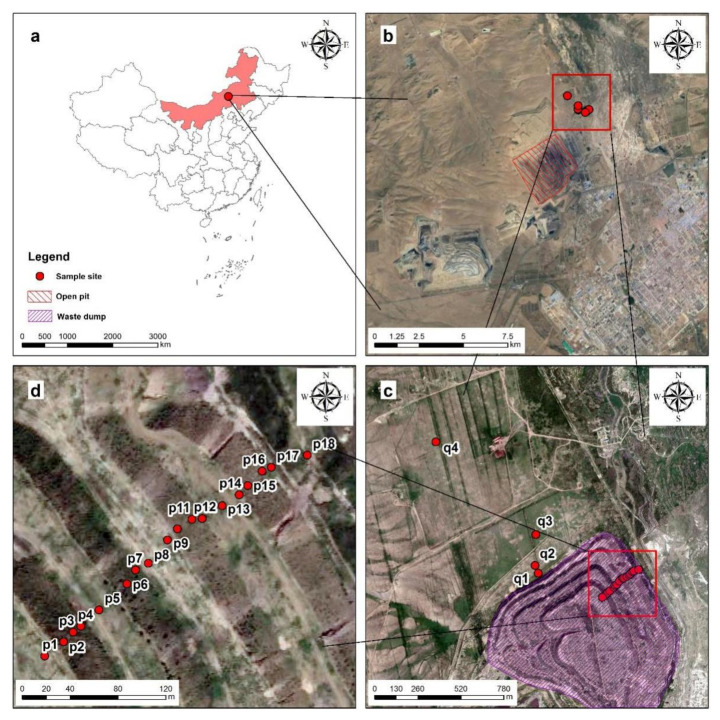
Location of study area and sampling points. (**a**) General location; (**b**) Shengli No.1 Open-pit Mine; (**c**) North dump (**d**) Research slope.

The study area was located at the Shengli No.1 Open-pit Mine and its surrounding areas in the Xilinguole grassland, Xilinguole League, Inner Mongolia Autonomous Region, northern China (43°54′15″–44°13′52″ N, 115°24′26″–116°26′30″ E), at an altitude of 960–1270 m (Figure 2a,b). Located in the mid-latitude westerly zone, it belongs to the mid-temperate semiarid continental climate, with warm and humid summer and cold and dry winter characteristics. The monthly mean temperature has a large span of −21.6 °C–19.0 °C. As for precipitation, it is 60–80% concentrated in the plant growing seasons, May-August, with an average annual precipitation of 294.9 mm and annual evaporation of 1600–1800 mm. In spring and winter, the wind is mainly from the southwest, with a wind speed of 2.1~8.4 m/s, an average wind speed of 3.5 m/s, and an instantaneous maximum wind speed of 36.6 m/s. There are three external mine dumps in this open-pit mine: the South dump, the North dump, and the Yanbang dump. In addition, there is an internal dump with a coverage of about 2.3 km × 1.1 km.

### 2.2. Sample Collections and Concentration Determination

The dump materials of different depths were randomly sampled by the five-point method in the pit. In order to analyze the spatial distribution of heavy metals present at the dump, a portable soil auger was used to sample the research slope at the northeast corner of the north dump. The four slope positions of the steps, including the platform, slope tip, middle slope, and slope toe, were drilled, and 1 m deep soil samples were extracted. There was a total of 18 samples packed in PVC pipes. Under the condition of ensuring homogeneity, each sample was cut into four sections with a depth of 0–25 cm, 25–50 cm, 50–75 cm, and 75–100 cm. Finally, 72 samples packed in polyethylene bags were obtained. The midpoint value represents the depth of the soil sample in this section (Figure 2c,d). Three topsoil samples with a depth of 0–25 cm at the bottom of the interior, south, and north mine dumps were drilled in the same way to compare dump-occupied soil with different discharge ages (3, 9, and 13 years). In order to eliminate human influence from other directions as much as possible, four soil interface samples with the depth of 0–10 cm located at 0 m, 50 m, 200 m, and 1 km in the natural area around the north dump were selected to explore the influence range of dump heavy metals (Figure 2c). Real-time kinematic (RTK) technology was used to record the coordinates of the sampling points. Each 1 kg sample was placed in a self-locking polyethylene bag and transported to the laboratory. The samples were first placed at room temperature for open-air drying in the laboratory. Then, the samples with roots and other plant materials removed were mortar ground, screened by 2 mm, and stored in a sealed sample bag for later use.

Each group of 0.5 g samples was digested on a heating plate at 180 °C for 10 h using the HNO_3_-HClO_4_-HF method. Then, inductively coupled plasma mass spectrometry (ICP-MS) was used to determine the concentration of Cr, Mn, Ni, Cu, Zn, Pb, and Cd [30,31]. Atomic fluorescence spectrometry was used to determine the concentration of As (AFS, HJ-680-2013, Ningbo, China) [28]. Quality assurance and quality control (QA/QC) can be achieved using reagent blanks, replicated samples, and standard reference materials. The standard reference materials (GSS-1~GSS-16) were used to control the measurement. The relative standard deviation (RSD) for the duplicated samples is <5%.

### 2.3. Statistical Analysis

Through the use of IBM SPSS 22.0 for Windows, the descriptive statistical analysis of the heavy metal concentration of the sample and the regression analysis of the heavy metal concentration of the soil interface with the distance was carried out. In addition, based on a one-way analysis of variance (ANOVA), the Kruskal–Wallis H test and Mann–Whitney U test were used to compare the differences in the concentration of heavy metals in different steps and slope positions.

### 2.4. Evaluation of Heavy Metal Pollution

In this paper, the geo-accumulation index method and the potential ecological risk index method were used to evaluate the heavy metal pollution of the mine dump.

As an essential parameter to distinguish the impact of human activities, geo-accumulation indexes of single metals were calculated to determine the pollution degree using Equation (1) [32,33]:

(1) $$I_{\mathrm{geo}}=\;\log_{2}{[C}_{n}{/(k\;\times \;B}_{n})]$$

where *C_n_* is the concentration of the metal examined in the samples, and *B_n_* is the geochemical background concentration of that metal. The background value of heavy metals in Inner Mongolia soil is taken here. *k* represents the background matrix factor due to lithogenic effects, usually 1.5. The geo-accumulation index consists of seven grades or classes. *I*_geo_ < 0 is class 0 (practically uncontaminated), 0 < *I*_geo_ ≤ 1 is class 1 (uncontaminated to moderately contaminated), 1 < *I*_geo_ ≤ 2 is class 2 (moderately contaminated), 2 < *I*_geo_ ≤ 3 is class 3 (moderately to heavily contaminated), 3 < *I*_geo_ ≤ 4 is class 4 (heavily contaminated), 4 < *I*_geo_ ≤ 5 is class 5 (heavily to extremely contaminated), and *I*_geo_ > 5 is class 6 (extremely contaminated).

The potential ecological risk (PER) index, proposed by Hakanson (1980) based on elemental abundance and release capacity, is widely used to evaluate the degree of heavy metal pollution in soils [34]. This method can reflect the impact of different heavy metal pollutants in a specific environment and the comprehensive impact of multiple heavy metal pollutants and quantitatively divide the potential ecological risk level [35]. The PER was estimated as Equations (2)–(4):

(2) $$C_{f}^{i}=\frac{C^{i}}{C_{n}^{i}}$$

(3) $$E_{r}^{i}{=T}_{r}^{i}{\;\times \;C}_{f}^{i}$$

(4) $$R_{I}=\sum E_{r}^{i}{=T}_{r}^{i}C_{f}^{i}{=T}_{r}^{i}C_{o}^{i}{/C}_{n}^{i}$$

where $C_{f}^{i}$ means the pollution factor of heavy metal *i*. *C^i^* is the measured concentration of heavy metal *i* in the sample, and $C_{n}^{i}$ is the corresponding background. The background value of heavy metals in the Inner Mongolia soil is taken here. $E_{r}^{i}$ is the monomial potential ecological risk factor of each heavy metal; $T_{r}^{i}$ is the toxic-response factor of each metal (Cd = 30, As = 10, Cu = Ni = Pb = 5, Cr = 2, Zn = Mn = 1) [36].

### 2.5. Spatial Analysis

**Figure 3:**
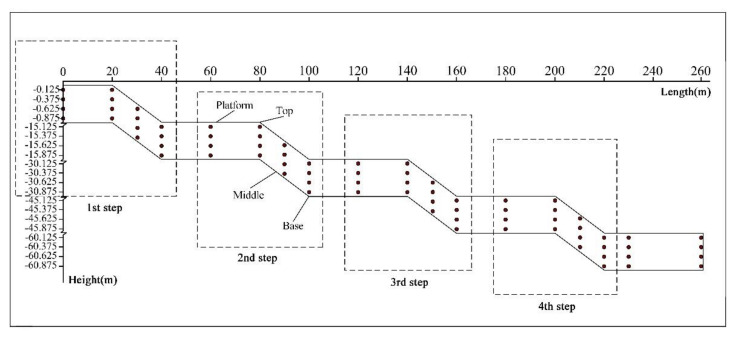
Sampling points distribution coordinate system of the soil interface of the mine dump.

The soil interface of the mine dump is a long and narrow strip. For the convenience of the display, the self-built irregular coordinate system based on the mine dump DEM was used to describe the spatial position relationship of sampling points. The abscissa was set according to the situation, and the ordinate was scaled up. The mine dump contains four steps in total, and each step is divided into four types of slopes (Figure 3).

The core of geostatistics is to determine the function rule of spatial position based on sampling points and infer the attribute value of unknown places [37]. The variation characteristics of spatial variables are based on the semi-variance function using Equation (5):

(5) $$\gamma \left(h\right)=\frac{1}{2N(h)}\overset{N(h)}{\underset{i=1}{\sum }}{[Z\left(X_{i}\right)\;{-\;Z(X}_{i}+h)]}^{2}$$

where *γ*(*h*) represents the value of the semi-variance function, *N*(*h*) represents the logarithm between locations at the distance *h*, and *Z*(*X*) represents the measured value of the variable *Z* at *X*. According to the classification criteria of [38], a spatial heterogeneity ratio *C_o_/*(*C_o_ + C)* of ≤25% means that variables have strong spatial autocorrelation; variables have medium spatial autocorrelation at 25–75%; variables have very weak spatial autocorrelation at ≥75%. The semi-variance function model was fitted with GS + 9.0. The data meet the normality test after collation and transformation. The Ordinary Kriging interpolation method in ArcGIS 10.22 was used to draw the spatial distribution figure of 8 typical heavy metals in the interface.

### 2.6. Soil Erosion Simulation

The Water Erosion Prediction Project (WEPP) simulated the erosion process of mine dump slope soil. Based on the mine dump and the surrounding area DEM, the climate generator CLIGEN generated a model weather data file based on the local weather data. The soil parameters were entered according to actual conditions. Assuming that the slope is a bare natural land with vegetation coverage of 0 and the surrounding area is grassland, the erosion results after 15 years were simulated [39].

### 2.7. Hyperspectral Inversion of Soil Interface’s Heavy Metal Concentration

**Figure 4:**
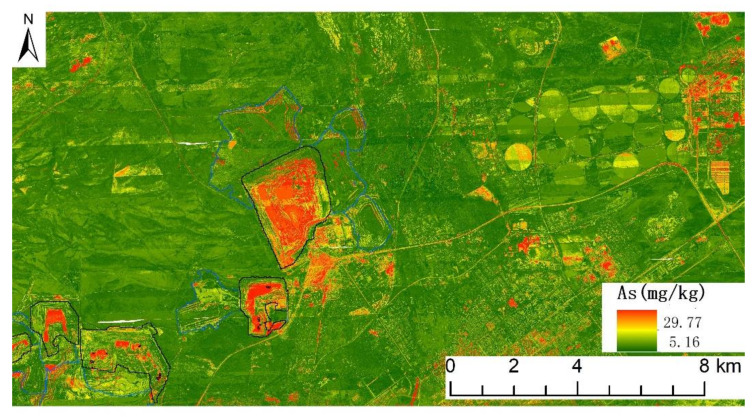
Hyperspectral inversion of arsenic concentration in the soil interface of the Shengli No.1 Open-pit Mine.

Through the combination of airborne hyperspectral remote sensing and field sampling, the spatial distribution of heavy metals in the soil interface was obtained. The grid method was used for preliminary layout and dense sampling of soil sample points in the Shengli mining area, with a grid size of 1 km × 1 km. The five-point method was used to determine the heavy metal concentration of the sample. Imaging spectrometers Hyspex and Headwall were used to collect hyperspectral images. The software with the sensor has the functions of radiation calibration and geolocation. Based on the high-resolution orthophoto, the registration of the hyperspectral data and the geometric precision correction of a single flight zone were carried out. The atmospheric radiative transfer model MODTRAN4 was used to perform atmospheric correction of navigational belts. Finally, the reflectance image data covering the entire study area was generated through the mosaic method based on geo-reference matching. According to the spectral reflectance of each sampling point extracted from the measured coordinates, the Competitive Adaptive Reweighted Sampling (CARS) algorithm extracted the characteristic bands of various heavy metals. Then, the Random Forest (RF) algorithm was used to construct an inversion model of topsoil’s heavy metal content. The spatial distribution of the soil interface’s heavy metals was obtained by applying each inversion model to aerial hyperspectral images (Figure 4) [20].

According to ArcGIS 10.22, the fan-shaped area within 400 m north of the mine dump was analyzed by a buffer zone. With a step length of 20 m, the inverted values of each buffer zone were extracted to obtain the law of the concentration of heavy metals in the soil interface with distance.

## 3. Results and Discussion

### 3.1. Evaluation of Heavy Metal Concentration and Pollution Degree

#### 3.1.1. Mine Dump Materials

The pollution effects of precipitation and irrigation water in the dump were eliminated by a sampling test. As a source of mine dump materials, open-pit coal mine waste rock-soil contains many heavy metal pollutants, which is the prerequisite that the mine dump will cause environmental pollution from long-term stacking [29,40]. The waste above the coal seam is fine sandstone, medium sandstone, gray claystone, shale, brown-gray claystone. Their average concentration of heavy metals is shown in the table (Table 1). Except for fine sandstone’s Mn, shale’s Cr and Mn, and brown-gray claystone’s Mn, Ni, and As, the concentrations of heavy metals in other layers are higher than the regional background value, indicating that the open-pit mine waste has the conditions to produce heavy metal pollution. The table clearly shows the origin strata of each metal. Among them, the contents of Cr, Mn, Ni, Cu, and Zn are higher in fine sandstone, medium sandstone, and gray claystone on the upper part. The contents of Cd and Pb in different strata are almost the same. The content of As is relatively random, mainly concentrated in the medium sandstone and shale layer.

#### 3.1.2. Mine Dump Soil Interface

**Figure 5:**
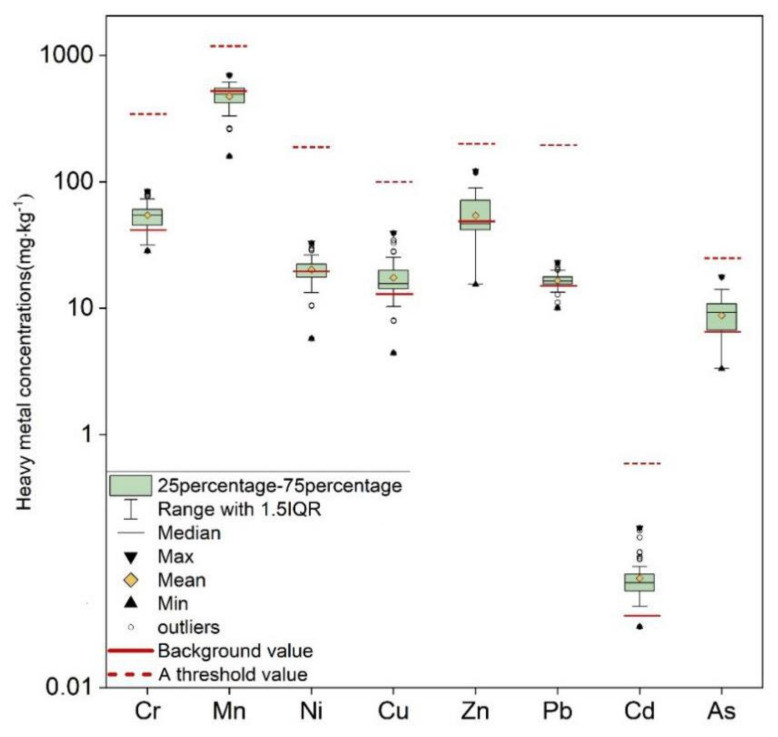
Characteristics of heavy metal concentrations in mine dump soil interface.

The soil interface is the primary channel for material transfer between the mine dump and the external environment. The results show the descriptive statistical data of heavy metal concentration in the mine dump soil interface (Table 2, Figure 5). Among them, the mean and median concentrations of Cr (58.24 mg·kg^−1^), Mn (500.17 mg·kg^−1^), Ni (23.58 mg·kg^−1^), Cu (18.11 mg·kg^−1^), Zn (55.40 mg·kg^−1^), Pb (16.82 mg·kg^−1^), Cd (0.09 mg·kg^−1^), and As (8.80 mg·kg^−1^) are all lower than the Chinese soil background value and the latest Chinese soil environmental quality standard limit value (GB15618-2018, GB36600-2018), indicating that they have little impact on agriculture and animal husbandry production, human health and the environment. However, the average concentration of Cr, Ni, Cu, Zn, Pb, Cd, and As of the other elements except Mn exceeds the background value of Inner Mongolia by 40.7%, 20.9%, 40.4%, 14.0%, 12.1%, 143.2%, and 39.7%, respectively, which may cause ecological harm [41,42,43].

Most elements such as Cr (36%), Mn (37%), Ni (52%), Cu (36%), Zn (40%), and Cd (118%) have high coefficients of variation (CVs) at 35%, showing a high spatial variation. Among them, Cd (118%) shows the most considerable variation, while Pb (15%) and As (34%) are 15–35%, showing moderate variation. In other words, the distribution of heavy metals in the mine dump topsoil is primarily affected by complex sources and random heaping [28].

**Figure 6:**
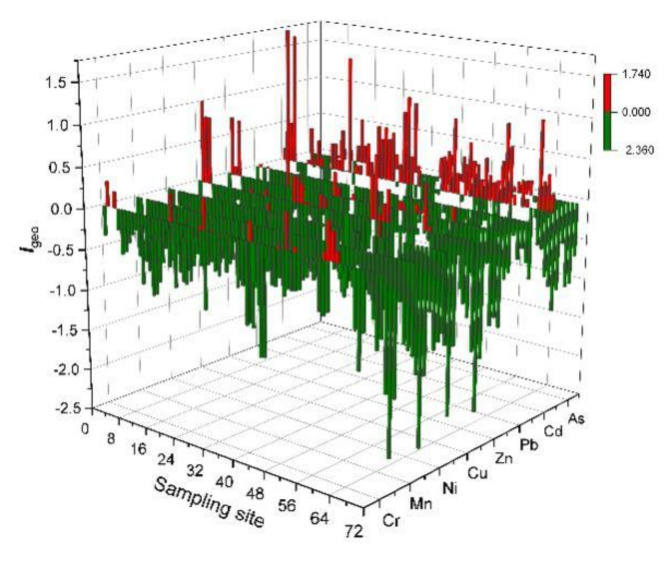
Geo–accumulation index value (*I*_geo_) of heavy metal in soil interface.

In order to identify the degree of heavy metal environmental pollution and assess its potential risks, the first thing is to conduct a detailed analysis of the geographic accumulation of heavy metals. As shown in the figure (Figure 6), the *I*_geo_ of Mn, Ni, and Pb is less than or equal to 0, indicating no pollution. Some of the *I*_geo_ of Cr, Cu, and Zn are between 0–1, indicating that there may be little pollution in some locations. The *I*_geo_ of Cd and As are basically in the range of 0–1, indicating slight pollution. The Cd on the top of some mine dumps reaches a moderate pollution level of 1–2. The figure shows the evaluation results of the potential ecological risk of heavy metals in the mine dump soil interface (Table 3). Among them, the risk indexes $E_{r}^{i}$ of single element Cr (2.62), Mn (0.90), Ni (5.16), Cu (6.74), Zn (1.11), Pb (5.45), and As (13.93) are all at low risk (<40). For the risk indexes $E_{r}^{i}$ of Cd (59.39), 4.17% of the samples are at low risk (<40), 79.17% of the samples are at medium risk (40–80), and 11.11% of the samples are at high risk (80–160). The potential ecological risk indexes *R_I_* of various heavy metals show that 77.78% of the samples are at low risk (<100), and 22.22% are at medium risk (100–200). Based on the above two evaluation methods, the heavy metal pollution degree of the mine dump soil interface is low. Although facing low potential ecological risks, it is necessary to pay attention to the ecological risks of elements such as Cd and As [44].

#### 3.1.3. Mine Dump Occupied Soil Interface with Different Discharge Ages

**Figure 7:**
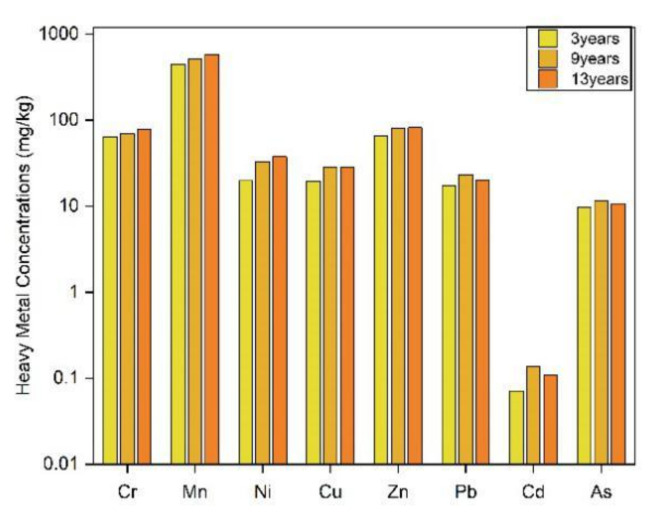
Heavy metal concentrations in dump occupied soil interface with different discharge ages.

The figure shows the concentration of heavy metals in the three mine dumps occupied soil interface with a discharge age of 3, 9, and 13 years in this mining area (Figure 7). According to the results, the concentration of heavy metals in the northern dump slope bottom occupied soil with a dumping time of 13 years is higher than the background value, and the pollution tends to migrate vertically. The concentration of eight typical heavy metals in the dump-occupied soil with a dumping time of fewer than three years is significantly lower than in the other two dumps. For the dump with a discharge time of 13 years, the heavy metal concentrations of Pb, Cd, and As are slightly lower, while the other elements are higher than in the dump with a 9-year discharge. According to the source analysis of heavy metals in the soil interface of the mining area, Pb and Cd come from automobile emissions and human activities. Considering the associated relationship between As and Ge, mining germanium in the southwest will lead to the heterogeneity of As distribution to a certain extent [28]. Since the 9-year dump is a demonstration area, it is significantly higher than the 13-year dump in human conservation, greening, and research. The two dumps have a similar discharge period, explaining the slightly higher Pb, Cd, and As concentrations. It can be concluded that the accumulation of heavy metals in the mine dump-occupied soil interface increases with the increase in the dumping age. In addition, the longer the heap discharge period, the more pronounced this trend.

### 3.2. Spatial Distribution of Heavy Metals in the Soil Interface of Mine Dump

#### 3.2.1. Spatial Heterogeneity

**Figure 8:**
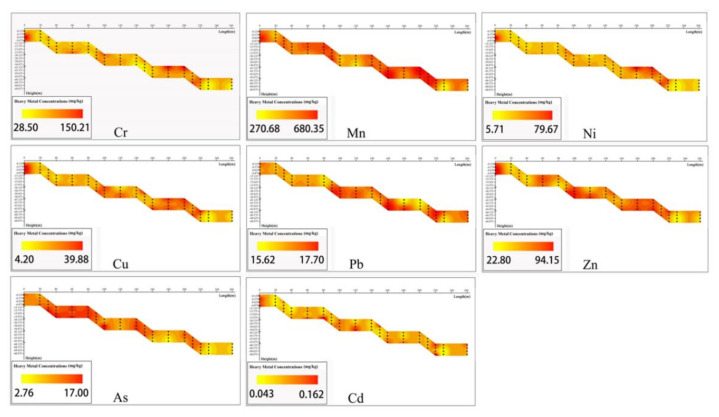
Heavy metal distribution of mine dump soil interface.

According to the heavy metal distribution figure of the mine dump soil interface (Figure 8) and the high nugget value (C_0_/(C_0_ + C) > 75%) and CVs of each heavy metal (Table 4), the heavy metal distribution of the interface has substantial heterogeneity. This may be due to the limitations of the early mine dump on the randomly mixed pile of waste and the current method of sampling large amounts of heterogeneous bodies [45]. The lower deposits caused by soil erosion on the slope will lead to the accumulation of heavy metals in the bottom steps of the slope.

#### 3.2.2. Horizontal Distribution

**Figure 9:**
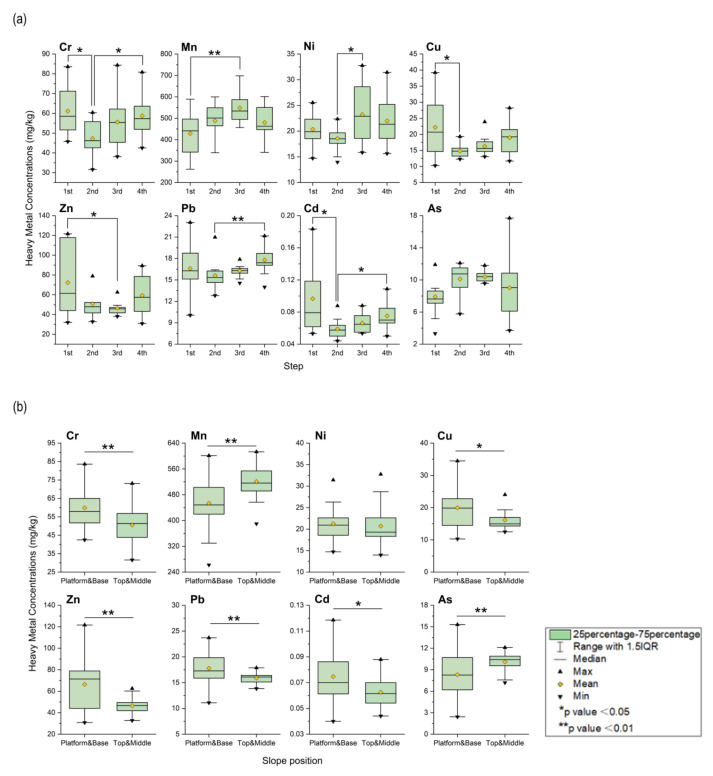
Horizontal distribution of mine dump soil interface. (**a**) Step; (**b**) Slope position.

According to the Kruskal–Wallis H test result in ANOVA, the boxplot graph is plotted (Figure 9a). The distribution of steps will affect the concentration of heavy metals. The concentration of heavy metals in the first step is generally high. The catchment area created by the retaining wall of the dump top plate will block water transportation, reduce soil erosion, and increase the resistance to heavy metal migration. Due to the artificial management of vegetation, the absorption of heavy metals will increase the content of heavy metals in rhizosphere soil. There are significant differences in the concentration of other elements except for As. As the number of steps increases, the concentration increases and reaches the maximum at the third and fourth steps. This may be caused by soil erosion deposited on the slope to the bottom step.

The Mann–Whitney U test results in ANOVA show that the boxplot graph is plotted (Figure 9b). The distribution of slope positions will affect the concentration of heavy metals. There are significant differences in the concentration of elements other than Ni in the slope positions, but the reason is not apparent and needs further exploration. Except for Mn and As, the contents of other elements in the flat plate and slope bottom are higher than the slope tip and middle slope. Among them, compared to the soil erosion areas such as the top of the slope and the middle slope, the essential quality of the soil and dump in the soil deposition areas of the flat plate and the slope bottom is more, and the vegetation grows better, which leads to the accumulation of heavy metals. The contents of Mn and As are opposite to those of other elements. Because the soil at the top of the slope and the middle slope is highly erosive, the occupied soil is scoured and stripped. The underlying substrate was confirmed to be mainly medium sandstone through field surveys. The concentration of heavy metals in the dump materials indicates that the Mn and As in the medium sandstone is much higher than in other formations, while the content of other elements remains stable [46].

**Figure 10:**
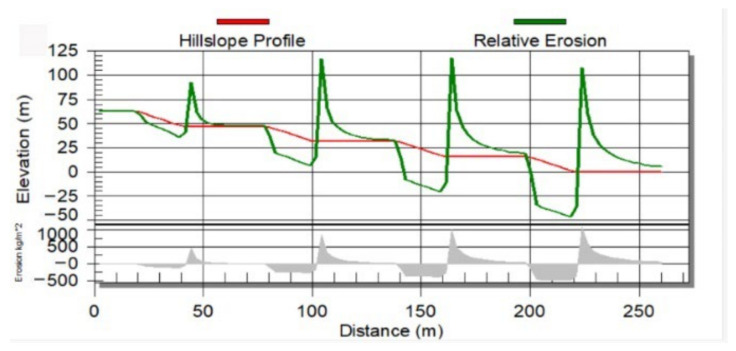
Soil erosion amount (the shaded part) simulation of dump slope.

The soil erosion simulation of the dump slope can sufficiently support the horizontal distribution law (Figure 10). Due to the particular continuous step structure of the dump slope, soil erosion of different degrees exists in each layer, and the sediment is deposited at the bottom and the platform below. The soil deposition amount of the lower step is more considerable than that of the higher step, which is about one time higher [47,48]. The soil deposition amount reaches the maximum at the bottom step of the dump, and there is a strong correlation between the soil deposition amount and the heavy metal concentration.

#### 3.2.3. Vertical Distribution

Combining the figure and the actual drilling measurement, the concentration of heavy metals in the vertical distribution showed an overall trend of low concentration in the upper layer and high concentration in the lower layer. This may be due to the intense infiltration caused by the high porosity of upper surface soil and the well-developed capillaries, and then heavy metals infiltrate with water [49,50]. In addition, partial plant absorption can reduce the content of heavy metals in the topsoil and matrix [51]. The aquiclude of the dump matrix is sandstone, and its permeability is between topsoil and claystone. Claystone and waste rock, as the water barrier, have low permeability. Under the influence of seepage flow, the migration of heavy metals from the upper layer to the lower layer is blocked and then accumulates in the aquiclude matrix. However, this rule is not apparent, with the specific manifestation of significant differences in several heavy metal concentrations of specific layers. Because of the early irregular discarding rules, mine dump failed to perform hierarchical discarding and had randomness, which resulted in substantial heterogeneity within the dump. In addition, existing technologies have difficulties sampling such enormous heterogeneity, which undoubtedly aggravates the randomness of the vertical distribution of dump heavy metal concentrations.

### 3.3. Distribution of Heavy Metals in the Soil Interface around Mine Dump

Based on the results of previous studies on heavy metals in the topsoil of this mining area, six typical pollution elements, Cr, Cu, Zn, Pb, Cd, and As, were selected for hyperspectral inversion [28].

**Figure 11:**
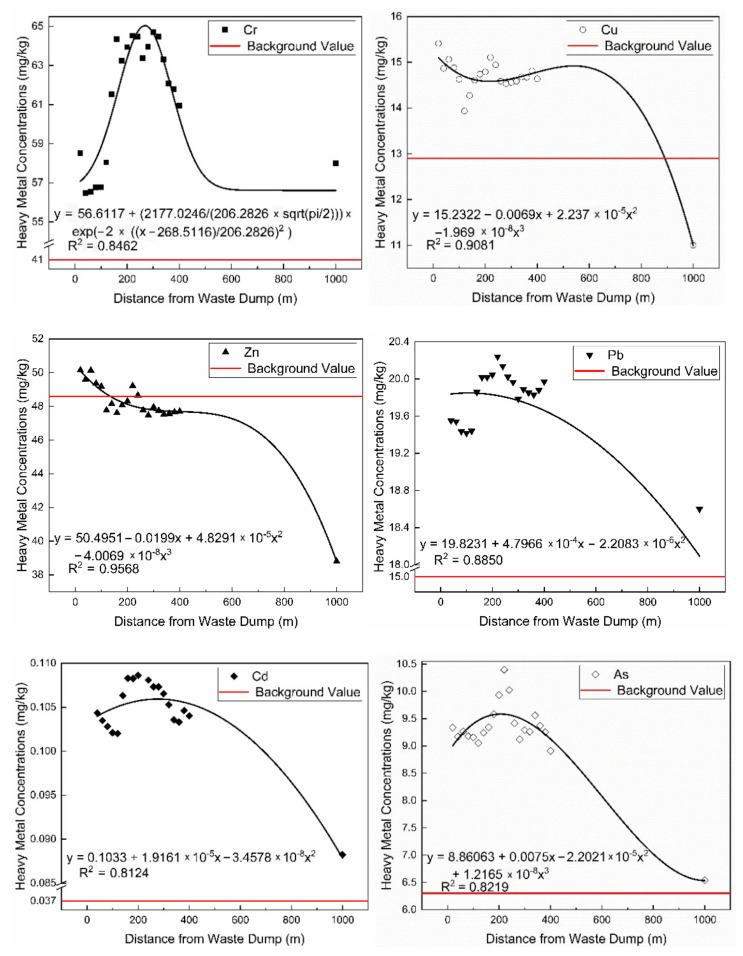
Regression analysis of soil interface heavy metal concentration and spatial distance.

The topsoil sampling results on the north side of the mine dump confirm the inversion results. The relationship between the concentration of heavy metals in the soil interface around the dump and the distance is found (Figure 11). In more detail, within 1 km, the concentration of heavy metals in the topsoil first decreases and then rises, reaching a peak at 220 m, and then continues to decrease with distance. Wind direction and soil erosion contribute to the phenomenon [52]. The concentrations of Cu, Pb, Cd, and As will drop sharply close to the background value after 1 km, while the concentration of Zn is lower than the background value after 200 m. If the concentration of Cr is to drop to the background value, a longer distance is needed.

## 4. Conclusions

Due to its concealment, open-pit coal mine dump heavy metal pollution lacks attention. Therefore, exploring its spatial distribution and migration characteristics is the critical point of revealing the existence of pollution and the essential prerequisite of analyzing and improving mine dump reclamation technology and preventing pollution. This study uses ANOVA and geostatistical spatial interpolation methods to describe heavy metal pollution’s spatial distribution and migration trend in a mine dump soil interface based on measured and hyperspectral remote sensing data.

By measuring the heavy metal concentration of dump-occupied soil with different dumping years, it can be found that dump heavy metal pollutants have time-series accumulation and longitudinal migration, which may cause pollution to the underlying stratum and water bodies. The soil interface is the material transport channel between the dump and the outside world, representing the distribution of heavy metals in the dump. On the one hand, dump heavy metal pollution distribution is strongly random due to disorderly piles. On the other hand, it is closely related to slope soil erosion. For example, all heavy metal elements converge at the bottom of the dump. Usually, the pollution in the lower part is higher than that in the upper part; the pollution in the lower step is higher than the upper step; the pollution in the soil deposition locations such as flat plate and slope bottom is higher than the soil erosion locations such as slope tip and middle slope. For the diffusion of dump heavy metal pollution to the surrounding area, the concentration of heavy metals first decreases with distance, then increases, and then continues to decrease and generally drops to the background value at about 1 km. Water and soil involved in soil erosion are important carriers for the migration of dump heavy metal pollutants. The relevant soil deposition area is where pollutants accumulate, which can be used to explore the area affected by pollution.

Therefore, control methods of mine dump soil erosion based on engineering and vegetation can also be used to improve heavy metal pollution. Pollution prevention and control should focus on the soil deposition area and adjust it according to the specific landscape and topography. This study provides a basis for the prevention and control of critical parts of mine dump heavy metal pollution and pollution migration path control, which is beneficial to improving the technology of dumping and reclamation, and protecting the grassland area’s ecology. The specific technical standards need to be further explored.

## Figures and Tables

**Table 1:** Lithology and heavy metal concentrations of mine dump materials.

Lithology	Heavy Metal Concentration (mg/kg)							
	Cr	Mn	Ni	Cu	Zn	Pb	Cd	As
Fine sandstone	62.70	469.00	26.30	32.80	93.90	19.60	0.13	21.60
Medium sandstone	63.50	537.00	26.90	32.90	121.00	21.80	0.11	41.60
Claystone (gray)	63.80	565.00	27.30	35.30	119.00	22.80	0.15	13.40
Shale	36.40	142.00	24.40	32.10	99.20	21.80	0.17	46.10
Claystone (brown and gray)	56.90	228.00	18.80	22.90	80.30	20.60	0.12	5.72
Inner Mongolia	41.40	520.00	19.50	12.90	48.60	15.00	0.04	6.30

**Table 2:** Comparison of heavy metal concentrations in mine dump soil interface with standard values.

	Heavy Metal Concentration (mg/kg)							
	Cr	Mn	Ni	Cu	Zn	Pb	Cd	As
Minimum	28.53	159.12	5.75	4.42	15.18	10.06	0.03	2.43
Mean	58.24	500.17	23.58	18.11	55.40	16.82	0.09	8.80
Median	54.87	492.61	20.36	15.95	47.93	16.33	0.07	9.13
Maximum	149.69	1623.77	79.69	39.22	121.64	23.70	0.80	17.70
Standard deviation	20.79	185.14	12.30	6.51	22.18	2.47	0.11	2.97
Coefficient of variation	0.36	0.37	0.52	0.36	0.40	0.15	1.18	0.34
Inner Mongolia	41.40	520.00	19.50	12.90	48.60	15.00	0.04	6.30
China	61.00	582.00	27.00	23.00	74.00	27.00	0.10	11.00
Chinese soil criteria	350.00	1200.00	190.00	100.00	200.00	200.00	0.60	25.00

**Table 3:** The potential ecological risk index value of heavy metals in soil interface (a) Ecological risk factor and assessment standards; (b) Potential ecological risk index and assessment standards.

$(a)\;E_{r}^{i}$	Risk Grade	Frequency Distribution%							
		Cr	Mn	Ni	Cu	Zn	Pb	Cd	As
$E_{r}^{i}$ < 40	Low	100.00	100.00	100.00	100.00	100.00	100.00	4.17	100.00
$40\;<\;E_{r}^{i}$ < 80	Moderate	0.00	0.00	0.00	0.00	0.00	0.00	79.17	0.00
$80\;<\;E_{r}^{i}$ < 160	High	0.00	0.00	0.00	0.00	0.00	0.00	11.11	0.00
$60\;<\;E_{r}^{i}$ < 320	Higher	0.00	0.00	0.00	0.00	0.00	0.00	0.00	0.00
$E_{r}^{i}$ ≥ 320	Serious	0.00	0.00	0.00	0.00	0.00	0.00	0.00	0.00
Range of ecological risk factor		1.38–4.07	0.00–1.34	1.47–8.40	1.71–15.20	0.32–2.50	0.00–7.68	24.58–148.69	5.29–28.10
Mean of ecological risk factor		2.62	0.90	5.16	6.74	1.11	5.45	59.39	13.93
$(b)\;R_{I}$		$R_{I}$ < 100	$100\;\leq \;R_{I}$ < 200	$200\;\leq \;R_{I}$ < 400	$R_{I}$ ≥ 400				
Frequencies		77.78	22.22	0.00	0.00				
Risk grade		Low	Moderate	High	Serious				
$\mathrm{Range}\;\mathrm{of}\;R_{I}$		27.31–197.80							
$\mathrm{Mean}\;\mathrm{of}\;R_{I}$		89.78							

**Table 4:** Semi-variance analysis of heavy metal in mine dump soil interface.

	Optimal Model	C_0_/(C_0_ + C)	R^2^	RSS
Cr	Gaussian	0.87	0.35	0.22
Mn	Gpherical	0.10	0.16	76.50
Ni	Eexponential	0.65	0.24	0.15
Cu	Spherical	0.10	0.65	0.07
Zn	Spherical	0.87	0.36	2.34
Pb	Linear	0.00	0.10	4.45 × 10^−3^
Cd	Exponential	0.97	0.04	2.17 × 10^−3^

## Data Availability

Data sharing is not applicable.
